# Diverse sources of normativity in open science and their implications for ethical governance

**DOI:** 10.1098/rsos.240480

**Published:** 2024-07-24

**Authors:** Kadri Simm, Jaana Eigi-Watkin

**Affiliations:** ^1^ Institute of Philosophy and Semiotics, University of Tartu, Tartu 51005, Estonia

**Keywords:** open science, ethics, challenges of open science, scientific pluralism, governance of science, philosophy of science

## Abstract

Over the past decade, open science (OS) has emerged as a global science policy and research initiative with implications for most aspects of research, including planning, funding, publishing, evaluation, data sharing and access. As OS has gained increasing prominence, it has also faced substantial criticism. Whether it is the worries about the equality of access associated with open-access publishing or the more recent allegations of OS benefitting those who act in the private interest without giving back to OS, there are, indeed, many potential as well as actual harms that can be linked to the practice of OS. These criticisms often revolve around ethical challenges and fairness concerns, prompting the question of whether a comprehensive ethical governance framework is needed for OS. This commentary contends that owing to the heterogeneous nature of the normative foundations of OS and the inherent diversity within scientific practices, a pluralistic and deliberative approach to governance is needed.

## 1. Advancing open science: the roots and the challenges

In very general terms, the objective of open science (OS) is to improve science—to make it more efficient (by sharing already existing data), transparent (and replicable), more inclusive and responsive to societal needs [1]. Clearly, these are all commendable objectives. Aspirational in nature, the roots of OS lie in a variety of different domains [2].

An important thread in the evolution of OS concerns access to research results. By now, the traditional academic publishing formats, where readers had to pay to access publications, have come to face fierce competition from various open-access publications [3,4]. The community-based approach to data sharing and collaboration can be traced back to the earliest days of software development as such, although the term free and open source software (FOSS) has been attached to those activities only retroactively since the 1980s [5]. Another significant branch of OS has its roots in citizen science that involves collaboration of non-scientists in scientific research outside of the traditional role of ‘research participant’. Citizen science is not new, spanning nearly a century, with citizens playing a vital role in environmental studies as data collectors and observers (e.g. in the Audubon ornithology projects [6]). In biomedical research, there has been a significant shift towards active patient participation and contribution from the 1990s onward (in orphan disease research, genetics, biobanking, etc.) [7].

Building on these developments, science policymakers [8,9] have undertaken coordinated efforts in recent decades to advocate for the democratization of science. As one example, the recent European Horizon 2020 *Science with and for Society* funding that focused, among others, on democratization and collaboration efforts between researchers and citizens, had its roots in the *Science and Society* funding that began in 2002 [10]. The objective is to enhance accessibility, transparency and promote direct engagement between science and society, as well as among key stakeholders [11]. For example, within the realm of biomedical research, this perspective advocates for shifting away from involving stakeholders, such as patients, solely in the final stages of product testing and approval. Instead, it emphasizes their inclusion in earlier stages of research, potentially even involving them in selecting research priorities or participating in funding decisions. By integrating stakeholders’ perspectives and expertise at earlier stages, the aim is to ensure that research aligns more closely with their needs and aspirations. Democratization of science has also been an object of increasing interest for philosophers of science and political theorists, whose arguments may reflect these developments and also show how democratization can be taken further [12,13].

While the above-mentioned developments are all rooted in well-justified concerns for the improvement of science, a variety of ethical concerns have also been voiced. Gold open access seems to have successfully switched the publication costs from readers to authors. This helps to take care of the problem of inaccessibility of research results (and it has been shown that open-access articles tend to have higher citations rates [14]). At the same time, it does little to address the concern that ultimately these costs are still going to come from the meagre funds of researchers, while publishers continue with high profits. In the context of global inequalities, the publishing costs are an especially large burden for researchers from low- and middle-income countries (LMIC). It is important to acknowledge that besides the conventional gold access model, which involves authors paying article processing fees and providing free access to readers, there are alternative options. Among them is green open access that allows for self-archiving of articles published in traditional journals, although this model may be subject to publishers’ embargoes. There is also platinum/diamond open access that is free for both authors and readers. The recent upward trend in journals adopting, or being established in, the latter category [15] is encouraging, as it suggests a positive response to those challenges. In addition, a number of behind-the-paywall or gold open-access journals have cheaper or no-cost arrangements in place for authors from LMIC. Yet the worry remains that open access, as currently practised, is still likely to further increase the inequalities within global research community. On the one hand, there is the concern that these developments may be insufficient to fully counteract the inequality perpetuated by academic publishing [16]. On the other hand, there is the more fundamental worry that regardless of the specific model of open access, data sharing is first and foremost going to be beneficial to those who have the resources to quickly capitalize on the data and publish results [2]. In other words, open access may foremost benefit those who are already well-off (e.g. have the funds and the prestige; [17]).

Ethical and methodological challenges have also been associated with citizen science practices [18–20]. One pertains to the potentially exploitative nature of some citizen science projects where participants have been contributing in terms of their time and resources but may not have been adequately acknowledged or compensated (or perhaps even aware of their participation) [21,22]. Participation of citizen scientists (in itself a heterogeneous concept [23]) may blur the well-established regulatory ethics boundaries separating researchers from research subjects, with possible implications for the integrity of research results [24]. Some researchers may also worry that the methodological expertise and research ethics training of professional scientists are often not available to citizen scientists. While professional scientists are certainly not immune to potential conflicts of interest, citizen scientists can often be explicitly motivated by advocacy issues. Finally, some socio-economic groups in society—typically, the more privileged—may be overrepresented among citizen scientists, with implications for what gets studied and for the representativeness of the data gathered [25].

It is important to stress that there are rebuttals to such concerns as well as developments towards appropriate standards and oversight [26–28]. However, the sheer heterogeneity of citizen science projects means that concerns are unlikely to be resolved once and for all.

The same applies to the issues for OS in general. Even though the overview above is certainly not an exhaustive list of ethical challenges associated with OS (and many of those challenges are being actively addressed), it helps to see why an approach to dealing with ethical issues in OS in a systemic and comprehensive manner—in other words, ethical governance of OS—appears desirable. In the remainder of the paper, we discuss critically the attraction and the limitations of such a unified approach.

## 2. Ethical governance of OS—where to start?

Ethical governance pertains to the explicitly moral aspects of processes, rules, values and principles of governance, and it is geared towards anticipating the ethical challenges in a systematic and coherent way rather than dealing with them as they might arise [29]. Guiding documents, i.e. various explicit articulations of values, principles, responsibilities and procedures, lie at the heart of ethical governance, although these need to be embraced and executed by institutions and organizations for implementation and accountability. Ethical governance is a broad church, both in terms of types and levels of entities dedicated to regulating the ethical (and other) aspects of their activities, as well as in terms of issues gathered under its accommodating umbrella. The focus of our article is not on documents providing guidance on particular domains of OS (e.g. DORA, CoARA on research assessment [30,31], the FAIR data principles [32], guidance and toolkits for citizen science and participatory science [33]) but rather on clarifying certain conceptual premises needed for comprehensive and coherent guidance.

An obvious starting point for thinking about the ethical framework for OS is, of course, research ethics. Over the past half-century, research has increasingly been governed by sets of formal ethical frameworks. Some have a broad scope, aiming to encompass research activities across all disciplines (such as the All European Academies European Code of Conduct for Research Integrity [34]), or the Singapore Statement on Research Integrity [35], although numerous influential documents focus on particular research fields or practices (e.g. the Belmont report on the protection of human subjects [36] or the Helsinki Declaration [37]).

Research ethics is a normative regulatory domain characterized by its set of values and principles. While it has some of its roots in the methodological requirements and practices of specific research fields (specifically, biomedical research), it has also evolved to serve as a means of overseeing professional research, particularly in terms of safeguarding the well-being of research participants.

Despite the diversity of scientific practices, there are certain values that can be considered applicable across all research endeavours. For example, the ALLEA Code highlights the principles of reliability, honesty, respect and accountability as fundamental to research integrity in general. It is fair to say that on that level of abstract generality, these values can well apply to OS. Additionally, OS highlights the values of transparency, reproducibility, new forms of collaboration, open access and fair evaluation of researchers [38,39]. However, it is important to note that research ethics might not be the only source for norms and values for OS. To further investigate the feasibility of an OS ethical framework, we need to look more closely at OS itself.

## 3. Open science and its many sources of normativity

We can think of OS as the point of convergence for several different sets of values. There are the values associated with different ‘roots’ of the movement, such as citizen science and FOSS. There are the traditional values associated with science as an institution (like Merton’s [40]) and, often, also science as a part of a democratic society [41]. There are the ethical values described in the previous section. Fecher and Friesike, in their influential analysis [2], show how this diversity finds expression in a limited number of more general ways to conceive of OS normatively. They structure OS as a broad field into five more or less distinct ‘schools of thought’, each with a different set of assumptions and objectives. The *pragmatic school* is interested in improving the efficiency of science by devising and facilitating new ways of collaboration between scientists. The *public and democratic schools* of thought, on the other hand, set the goals for OS from the vantage point of broader social benefits and inclusion (in terms of involving citizens in research processes as well as making research results accessible and understandable). The *infrastructure school* focuses on improving research infrastructures and processes, and the *measurement school* is concerned with alternative ways of measuring scientific outputs and impact.

What follows from this descriptive mapping of OS for the prescriptive ethical governance of OS? It seems clear that OS contains a number of sources of normativity—what counts as good or responsible OS—and this is in itself one root of challenges, as in satisfying some objectives, it may be seen as failing others. There are, in that sense, contradictions within the ideals of OS itself, and even a perfect implementation of one set of goals is likely to ignore or even work against the aims of another set of goals, anchored in a different school of OS thought or other normative accounts of science.

Düwell has highlighted some of these tensions and a need for more nuance and clarity within the OS objectives of social engagement and democratization of research processes (the democratic and public schools). Certain democratic ideals of engagement and ‘direct responsiveness to societal challenges’, if seen as prescriptions from society to science, would work against the freedom of academic research, and these requirements applied to research are likely to be short-sighted [42].

Fernández Pinto [43] has argued that since the majority of global research (at least in terms of expenditure) is private, the OS movement’s focus on opening up taxpayer-funded research leaves privately funded research off the hook. The OS policy objective of making publicly funded research results open for commercialization and accessible to private industry is worrisome from the perspective of the Mertonian communal ethos of science. Also, commercial research has a problematic track record of transparency and accountability [43]. This criticism is taken further by scholars who have linked the OS movement more explicitly to the so-called neoliberal knowledge economy. They are pointing to the many ways in which OS practices end up supporting further commercialization of research, whether by continuing to cash in on research publishing or by reaping the fruits of the open data movement. The worry here is that OS has paid insufficient attention to corporate interests and the many ways in which power is hidden in contemporary knowledge production [44]. This suggests that proponents of the OS discourse are either naive or worse, complicit in the continuing commercialization of research and the worsening of social global inequities in research [45].

It is important to stress that there are ongoing developments aiming to counteract these tendencies, seen as undesirable from the point of view of the *public* and *democratic* schools of thought. For example, the continuous development (increasingly pushed by research funding organizations) of open-access publishing options (including low-cost or no-cost options) has offered more choices and opportunities for all researchers. Another example, an alternative route to the proprietary commercial model of research is characterized by the proprietary but non-commercial options long established in software development, particularly in fields like scientific computing [46]. Crucially, however, at least some of these trends are not necessarily problematic from the perspective of the *pragmatic* and *infrastructure* schools of OS if they result in a more efficient science (although *efficient for whom* might well be asked). From the point of view of these schools, it is the spending on alternative forms of open access or support for other alternatives that may be counterproductive.

Citizen science offers another locus for examining the potential paradoxes of OS norms. Haklay *et al*.’s [47] study of the contours of citizen science demonstrates how comprehensive and demanding the criteria might be for a collaboration to be considered citizen science proper. The aspirational idea of citizen science epitomizes ‘a new social contract’ [19] of empowerment that begins with community-embedded schooling [48]. It envisages citizen scientists as active and systematic contributors and participants throughout the various stages of the research project, including substantive contributions, real decision-making power, opportunities for education and learning and a voice in commercialization of results. Indeed, all these aspects are important if the goals of *democratic and public schools* of OS are to be taken seriously. However, it is yet to be seen whether these trends would work for or against making science more effective (*pragmatic* school), or how these time- and resource-thirsty engagements could be standardized and accounted for to achieve the goals of the *measurement* school.

While the old Mertonian ideal proposed that within the ethos of science, the scientific and democratic ideals coincided, the broadening of scientific practices and objectives under the umbrella of OS is challenging this assumption and the convergence of Mertonian and OS principles has been called into question [49]. At its core, research aspires towards meritocracy [50] and although there are ample data that the practice of science often fails to live up to these ideals, researchers commonly continue to uphold them [51]. At the same time, ideals of meritocracy potentially clash with the ideals of inclusion and democratic decision-making that are prominent, for example, within citizen science.

## 4. Science and its many varieties of plurality

As we have argued, different ethical and political objectives for OS constitute one source of normative diversity. Another important source is normative epistemic considerations—considerations related to science’s knowledge-producing practices. In philosophy of science, which analyses them, various forms of pluralism have been increasingly influential [52–54]. Philosophers of science have been acknowledging, and drawing attention to, pluralities that characterize science on different levels. In science(s), there are different aims, values, theories and approaches; different accounts of the same phenomena; different explanations, methods, models, classifications, etc. Such plurality exists both between scientific disciplines (chemistry is different in many respects from physics) and within the same discipline (chemistry itself is in many respects pluralistic) [55]. Collaborations between academic researchers and parties outside of academia—the increasingly important transdisciplinary research—also add to the diversity of aims, values and practices [56].

One way in which such diversity has implications for the governance of science is through the issue of evaluation of research. What counts as appropriate methods and high-quality data depends on the aim of research [57]. In the situation where there is a plurality of different legitimate aims for research, approaches to research evaluation need to take these differences into account. A mismatch between the aims and values of a discipline on the one hand and the assumptions involved in quality evaluation on the other is a worrying possibility. Such a mismatch may result in valuable knowledge not being recognized as such or not being produced in the first place [58]. With OS, this possibility becomes more pressing and more widespread, since potentially many more aspects of science will be open to much wider evaluation by various parties. Leonelli [59] shows how this may already be happening in the context of OS on the example of biology. When certain advanced technologies are assumed to be the gold standard for producing high-quality biological data, researchers for whom producing such data is both unfeasible and irrelevant may be discouraged from making their own data open and may fail to benefit from the data shared by others. This is concerning both epistemically (loss of valuable knowledge) and ethically (growing unfairness).

## 5. What to do? Pluralist settings

The UNESCO recommendation on OS [60] specifically calls for the promotion of a shared understanding of OS, and the recent policy addition to the European Code of Conduct on Open Science [38] similarly provides a set of principles to guide OS participants, primarily inspired by the research ethics and integrity discourse. Despite the tensions between different schools of thought within OS, a shared understanding of OS might indeed be possible, albeit on the level of abstract values and principles. How should one proceed from there? Pluralist guidance, while appropriate, is also one where the multitude of relevant ethical values and principles requires constant reflection, vigilance, transparency and context-sensitivity in figuring out what applies where, when and why.

With a movement as broad and complex as OS, it is evident that certain practices within OS may yield advantages for specific stakeholder groups while inadvertently disincentivizing or potentially harming others. The plurality of science and the variety of OS discourses do not only mean that sometimes the goals are different, but that they can be incompatible. If one is to view OS as a pluralist undertaking—the not-so-seamless integration of diverse sets of objectives for the very epistemically diverse and pluralistic undertaking of science—it is understandable that particular goals might be prioritized in particular contexts and others at other times.

What is important, in terms of the ethical governance of OS, is to be transparent and reflective about the existence of diverse goals within OS and to acknowledge the necessity as well as the duty to balance these sometimes paradoxical aims, at least in the long term. Adopting a pluralistic approach also aligns well with the prevailing discourse in contemporary philosophy of science.

In more practical terms, our recommendations concern, first, the way the guiding documents and policies are formulated. We recommend being explicit about the aims and values a given document/policy intends to promote and about other potentially relevant aims and values it may fail to advance or for which it may even be counterproductive. Such an explicit acknowledgement of plurality also needs to be a part of public communication about OS.

Second, our recommendations concern the education of scientists, citizen scientists and other practitioners of OS. The plurality of norms, aims and values of OS needs to be communicated to them as a part of their education in OS. In addition, it is important to include the development of the awareness of, and the ability to reflect upon, potentially relevant aims, values and stakeholders as one is choosing the course of action when doing research. One example of a teachable approach for doing so is the framework of moral imagination developed by philosopher of science Matthew Brown [61]. This method aims to facilitate the realization of research goals by combining considerations of ethical, epistemic and stakeholder concerns with the imaginative exploration of diverse options of achieving research goals and can be applied individually or in collaborative settings with colleagues.

It must be acknowledged that OS in its current form is not well positioned to solve or even alleviate a number of ethical wrongs in global research. While the issue of inclusion of LMIC researchers has at least somewhat progressed beyond mere exclamations of solidarity (e.g. lower article publishing charges (APCs), benefit-sharing agreements), the current operational area for ethical OS continues to leave private research off the hook and is yet to engage thoroughly with the novel challenges of generative AI [62]. Ultimately, it is the responsibility of those with more power and resources (e.g. funding agencies, high-income country researchers and research institutions) to take a broader view of the challenges faced by OS practitioners globally and make sure that OS continues to flourish as a comprehensive and fair initiative.

## Data Availability

This article has no additional data.
